# Identification of *GA20ox2* as a target of ATHB2 and TCP13 during shade response

**DOI:** 10.3389/fpls.2023.1158288

**Published:** 2023-04-21

**Authors:** Ora Son, Chaoyue Zhang, Xiaoyu Yang, Le Thi Duc, Yoon-Sun Hur, Kyoung Hee Nam, Soon-Young Choi, Choong-Ill Cheon, Sunghan Kim

**Affiliations:** ^1^ Department of Biological Science and Institute of Women’s Health, Sookmyung Women’s University, Seoul, Republic of Korea; ^2^ Department of Systems Biology, Yonsei University, Seoul, Republic of Korea

**Keywords:** ATHB2, TCP13, shade avoidance syndrome, *GA20ox2*, GAF1

## Abstract

The shade avoidance syndrome (SAS) is a collective adaptive response of plants under shade highlighted by characteristic phenotypes such as hypocotyl elongation, which is largely mediated by concerted actions of auxin and GA. We identified ATHB2, a homeodomain-leucine zipper (HD-Zip) domain transcription factor known to be rapidly induced under shade condition, as a positive regulator of GA biosynthesis necessary for the SAS by transactivating the expression of *GA20ox2*, a key gene in the GA biosynthesis pathway. Based on promoter deletion analysis, EMSA and ChIP assay, ATHB2 appears to regulate the *GA20ox2* expression as a direct binding target. We also found that the *GA20ox2* expression is under negative control by TCP13, the effect of which can be suppressed by presence of ATHB2. Considering a rapid induction kinetics of *ATHB2*, this relationship between ATHB2 and TCP13 may allow ATHB2 to play a shade-specific activator for *GA20ox* by derepressing a pre-existing activity of TCP13.

## 1 Introduction

Plants make use of light for sustaining their lives through photosynthetic processes and transduction of light signals. In crowded plant canopies, the lower layers of plants receive reduced amounts of photosynthetically active radiation (PAR, 400-700 nm), over a specific range of wavelengths of light in particular. The upper leaves preferentially absorb the red (R) and blue (B) wavelengths of sunlight and transmit and reflect the far-red (FR) wavelengths (Casal, 2013; Roig-Villanova and Martínez-García, 2016; Fernández-Milmanda and Ballaré, 2021; Pierik and Ballaré, 2021). This reduces the R:FR ratio of light in shaded areas, as a result, and induces a series of responses that enable the shaded plants to capture unfiltered light. Primarily, it promotes the elongation of petioles, internodes and hypocotyls and the hyponastic growth of leaves, while it inhibits the expansion of cotyledons and mature leaves. In addition, early flowering and reduced seed yield as well as reduced defense against plant pathogens are observed. These effects are collectively known as the shade avoidance syndrome (SAS). Although the SAS is a strategy for the shaded plants to adapt to natural environments, it is at the same time considered to be detrimental to crop productivity as it diverts resources for growth to support stem growth (Pierik and Testerink, 2014; Wang et al., 2020; Fernández-Milmanda and Ballaré, 2021).

The roles of plant hormones on the SAS have been extensively examined as they the key mediators for the most developmental processes of plants (Bou-Torrent et al., 2014). Auxin, a principal regulator of cell elongation well documented in the case of hypocotyl elongation through relevant transcription factors, has been identified as the major player modulating the hypocotyl elongation in response to shade as well (Tao et al., 2008; Oh et al., 2014; Chaiwanon et al., 2016; Procko et al., 2016; Yang and Li, 2017; Reed et al., 2018; Ma and Li, 2019). Tao et al. (2008) described TAA1, an enzyme which catalyzes the first step in auxin biosynthesis pathway, is rapidly deployed to synthesize auxin at the high levels to initiate the changes in plant associated with shade avoidance. Under the prolonged shade condition, auxin contents decrease while the auxin sensitivity increases, resulting in the enhanced auxin perception and signaling (Pucciariello et al., 2018).

A number of evidence indicate that gibberellins are also involved in the shade-induced changes in plants (Casal, 2013). FR-irradiated epicotyls exhibited higher amount of GA_1_ than R-irradiated ones (Martínez-García et al., 2000). Petiole elongation by a FR stimulus was reduced in *AtGA20ox2*-silenced plants (Hisamatsu et al., 2005), and petiole elongation rates coincided with RGA breakdown, showing the strong correlation of GA with shade responses (Djakovic-Petrovic et al., 2007). Shade treatment for 4 h resulted in increased levels of auxin but rather decreased levels of BR, and mildly increased levels of GA, while a prolonged shade for 24 h exhibited increased levels of GA but the same level of auxin and BR as non-shaded seedlings, showcasing a complex dynamics of hormone balance during the entire stage of shade response, which may be interpreted as that in the long-term shade treatment gibberellins might be the one contributing to the hypocotyl elongation rather than auxin (Bou-Torrent et al., 2014). GA has been known as an essential hormone for hypocotyl and stem elongation (Yamaguchi, 2008), with its major contribution recognized in shade responses as described above, but how shade increases active GA remains obscure.

*Arabidopsis thaliana* homeobox 2 (ATHB2), a member of the homeodomain- leucine zipper family II (HD-Zip II), is induced by a low R:FR (Carabelli et al., 1993; Carabelli et al., 2013). Its HD-Zip domain has been identified to bind to a 9-bp dyad symmetric consensus sequence (Sessa et al., 1993). ATHB2 was demonstrated to be a direct target of PIF4/PIF5, major transcriptional regulators of SAS (Kunihiro et al., 2011; Favero et al., 2020; Zhang et al., 2020). In addition, PIF7 was also reported to strongly bind to the upstream region of *ATHB2* (Willige et al., 2021). Overexpression of *ATHB2* resulted in longitudinal cell elongation in hypocotyls (Steindler et al., 1999), while mutant analyses of *ATHB2* indicated that ATHB2 and its close paralogues (ATHB4 and HAT3) together control apical embryo (Turchi et al., 2013). In *athb-2* mutant, *YUCCAs*, genes encoding a rate-limiting enzyme for auxin biosynthesis, were still found to be fully induced by low R:FR treatment, indicating that ATHB2 is not required for auxin biosynthesis during shade responses (Müller-Moulé et al., 2016). Rather, *ATHB2* induction resulted in reduced expression of some auxin biosynthetic genes including *YUCCAs* (He et al., 2020). The three HD-Zip II protein genes, *ATHB2, ATHB4* and *HAT3*, exhibit a common functional characteristic including being inducible by a low R:FR and conferring shade response-specific phenotypes in transgenic plants overexpressing them (Steindler et al., 1999; Sorin et al., 2009; Turchi et al., 2015). ATHB2, ATHB4, and HAT3 were also shown to interact with TCP13, TCP17 and TCP5, members of the CIN-like TCP family which are transcriptional regulators of plant development in a reciprocal manner (Martin-Trillo and Cubas, 2010; Hur et al., 2015; Lopez et al., 2015), implying their functional association as modular regulatory component in coordinating the shade avoidance response, since these TCPs have been also implicated in the process. However, how exactly the function of ATHB2 is played in shade avoidance, especially in regulating hypocotyl elongation in response to shade, remain unknown.

In the present study, we explored a possibility of ATHB2 as a regulator of GA biosynthesis for promoting hypocotyl elongation under shade condition. We found that rapid induction of *GA20ox2* in response to shade could be attributed to ATHB2-mediated transcriptional activation as evidenced from *GA20ox2* expression analyses made after protoplast transfection as well as in transgenic plants expressing *ATHB2*. The stimulatory function of ATHB2 on the expression of *GA20ox2* was found to be tightly associated with a *cis*-regulatory sequence element present on the promoter of *GA20ox2* and a direct binding of ATHB2 was also demonstrated by EMSA and ChIP analyses, suggesting that *GA20ox2* is likely to be a direct target of the ATHB2-mediated transactivation. On the contrary, we found that TCP13 functioned as a negative regulator of *GA20ox2* expression and the site of its action on the promoter is distantly located from that of the ATHB2, but its effect is still affected by the presence of ATHB2, suggesting ATHB2 and TCP13 are playing opposite roles in regulating *GA20ox2* expression and subsequent GA synthesis under shade condition to provide a balance in coordinating the hypocotyl elongation response.

## 2 Materials and methods

### 2.1 Plant material and growth condition

*Arabidopsis* seeds (Col-0) were surface-sterilized in 70% ethanol and 0.01% Tween-20 for 15 min, rinsed twice in 95% ethanol, and once again in 100% ethanol. *Arabidopsis* plants were grown on half-strength Murashige and Skoog (MS) medium supplemented with 1% sucrose and 0.8% plant agar in a growth chamber at 22°C under long-day conditions (16-h light/8-h dark photoperiods) at 50 µmol m^-2^ s^-1^.

### 2.2 Plant treatments

Seven-day-old seedlings grown under continuous white light (W; 24 µmol m^-2^ s^-1^, R:FR ratio > 2.1) were treated with simulated shade (W+FR; R:FR ratio of 0.05, W enriched with supplementary FR provided by QB1310CS-670–735 LED hybrid lamps of Quantum Devices Inc.) in time course (0 h, 1 h, 2 h, 3 h, and 4 h), and then used for RNA extraction. Four-day-old seedlings grown under continuous white light (W) were treated with simulated shade (W+FR) for 3 days, or white light for 3 days as a control, and then used for the observation of shade avoidance phenotypes. For the dexamethasone (DEX) treatment, 7-day-old seedlings were transferred to the liquid MS medium containing 1 or 10 μM DEX, or 0.01% ethanol alone during the indicated times (Hur et al., 2015).

### 2.3 Vector construction and plant transformation

To generate transgenic plants containing *GVG::ATHB2-GFP*, *ATHB2-GFP* fusion fragment was amplified by PCR using a vector containing *P35S::ATHB2-GFP* (Hur et al., 2019) as template, and cloned into the pTA7002 vector (Hiratsu et al., 2003) using *Xho*I and *Spe*I. The resulting vector was introduced into *Agrobacterium tumefaciens* strain GV3101, and used for floral dip transformation of *Arabidopsis* (Col-0). The primers used for making constructs were shown in **Supplementary Table S1**.

### 2.4 Measurement of hypocotyl lengths

Five-day-old seedlings were exogenously treated with MOCK solution, 4 μmol 3- nitropropionic acid (NPA), and 1 μmol paclobutrazol (PAC) for 24 h before transferring to growth chambers with different light conditions for 5 days. Hypocotyl lengths were measured with Image J software (http://rsb.info.nih.gov/ij) using the pictures of seedlings (Ince and Galvão, 2021). All data were statistically evaluated using one-way ANOVA.

### 2.5 Transient gene expression assay

Protoplast isolation and transfection from *Arabidopsis* leaves were performed according to Yoo et al. (Yoo et al., 2007). Plasmid DNAs were extracted using cesium chloride (CsCl)/ethidium bromide (EB) centrifugation. Protoplasts (1 x 10^4^) were transfected with a total of 10 μg of plasmid DNAs and incubated for 16 h in the dark. Protoplasts were harvested and subsequently used in real-time quantitative PCR or luciferase assay.

### 2.6 Real-time quantitative PCR

RNA was extracted from leaves or seedlings using RNeasy plant mini kit (Qiagen) and reverse transcription of RNA was performed using Moloney Murine Leukemia Virus (M- MLV) reverse transcriptase (Promega). Real-time quantitative PCR was performed with SYBR Green PCR Master Mix (Takara) using a LightCycler 96 (Roche, Mannheim, Germany). mRNA levels were normalized with *EF1α* and *ACTIN2*. The primers used for real-time quantitative PCR were shown in **Supplementary Table S1**.

### 2.7 Dual luciferase assay

The cauliflower mosaic virus 35S promoter (*35S pro*)::*ATHB2-GFP* or *35S pro::TCP13- GFP* or *35S pro::TCP13-VP16* were used as effector plasmids. Different lengths of upstream region of *GA20ox2* (1.5-kb, 1.0-kb, 0.5-kb, 0.2-kb, respectively) were fused to luciferase gene to make *GA20ox2 pro::LUC* as reporter genes, and *Renilla* luciferase (*35S pro::RLUC*) was used as internal control. Mutagenized versions of 1.5-kb upstream region of *GA20ox2* (one at ABS1 and the other at ABS2) were fused to *LUC*; ABS1(m) and ABS2(m). *Arabidopsis* protoplasts were isolated and transfected by the polyethylene glycol (PEG)-method as previously described (Yoo et al., 2007). Luciferase activity was measured with a dual-luciferase reporter assay system according to the manufacturer’s instruction (Promega) in a SpectraMax i3X microplate reader (Molecular Devices). Firefly luciferase (LUC) activity was normalized with *Renilla* luciferase (RLUC) activity.

### 2.8 EMSA

Electrophoretic mobility shift assay (EMSA) was performed using a LightShift^®^ Chemiluminescent EMSA Kit (Thermo Fisher Scientific) according to the manufacturer’s instruction. Oligonucleotides (31-mer) flanking the ABS1 were labeled by 3’ end biotin, and unlabeled oligonucleotides were used as competitors as shown in **Table S1**. His- tagged ATHB2 protein was isolated from *E. coli* transformed with *ATHB2*-containing pET-28a using Ni-NTA agarose (Qiagen) and further purified using Amicon^®^ Ultra-15 centrifugal filters (Millipore).

### 2.9 Yeast two-hybrid assay

Yeast strain and methods used in the yeast two-hybrid assay were essentially the same as in Son et al., 2016. TCP13 partial cDNAs were subcloned into pGBKT7 as baits, while GAF ORF cDNA was subcloned into pGADT7 as a prey, respectively (Clontech Inc.). Primers used were shown in **Supplementary Table S1**.

### 2.10 Chromatin immunoprecipitation (ChIP) assay

For quantitative chromatin immunoprecipitation (ChIP)-PCR assays, 10-day-old seedlings with either *GVG::ATHB2-GFP* or *GVG::3HA-GFP* were harvested after treating with10 μM DEX for 48 h. ChIP was performed as described by Gendrel et al. (2002). Briefly, leaves were cross-linked with 1% (v/v) formaldehyde solution by vacuum infiltration for 10 min. Chromatin complexes were sonicated with a Ultrasonic Processor (VC-505, SONICS) in a nuclei lysis buffer (50 mM Tris-HCl, 10 mM EDTA, 1% SDS, 1 mM PMSF, 1 X Protease inhibitor cocktail, pH 8.0), and the protein-DNA complexes were incubated with an anti-GFP antibody (ab290, Abcam) overnight at 4°C. Protein A/G PLUS-Agarose (Santa Cruz Biotechnology) was added and incubated for 2 h. The fractions were washed and eluted with elution buffer (1% SDS and 0.1 M NaHCO_3_) at 65°C for 30 min. The fractions were reverse cross-linked with NaCl (final concentration 0.2 M) at 65°C overnight. The precipitated DNAs were purified using QIAquick PCR Purification Kits (Qiagen), and analyzed using the real-time quantitative PCR. The primers were designed to amplify fragments within *GA20ox2* promoter (**Supplementary Table S1**).

## 3 Results

### 3.1 GA biosynthetic genes are rapidly induced upon shade treatment

**Figure 1:**
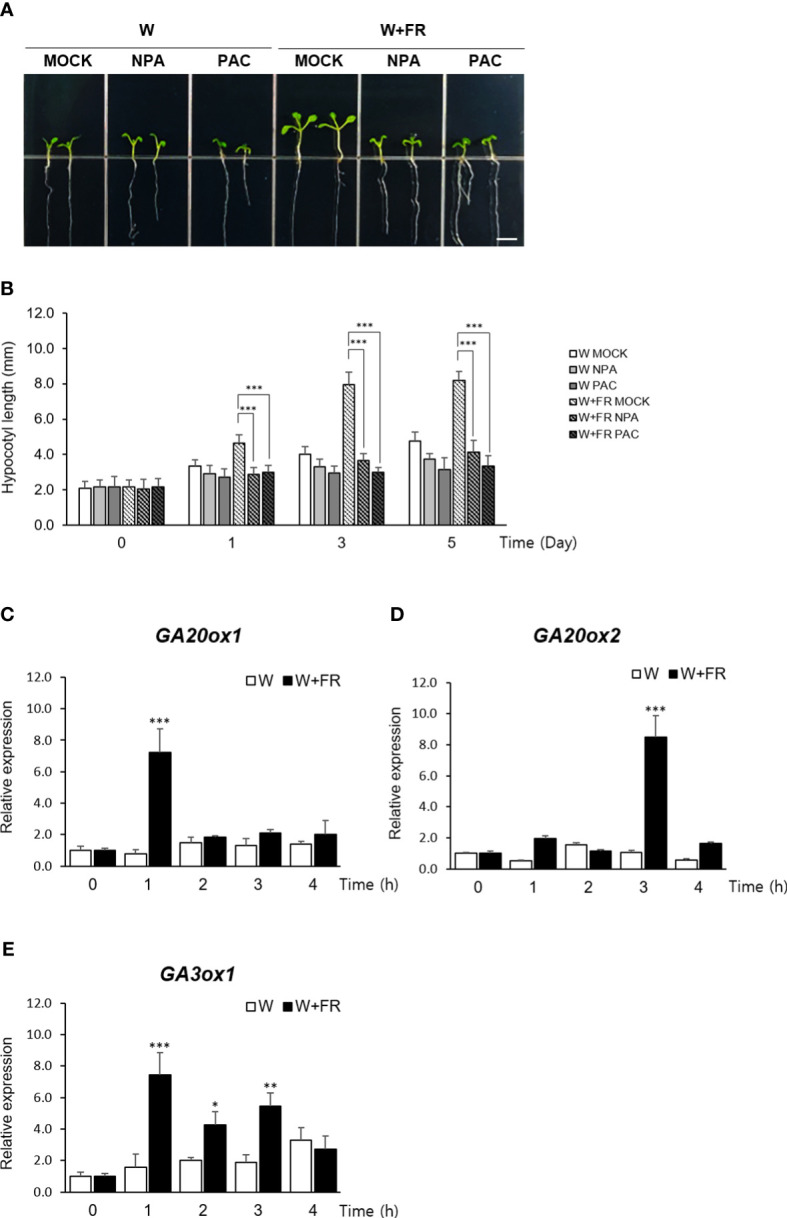
Expression of GA biosynthetic genes under the shade condition. **(A)** Effects of auxin transport inhibitor NPA or GA biosynthetic inhibitor PAC on hypocotyl lengths of *Arabidopsis thaliana* under shade condition was observed. Light condition was as follows: W, continuous white light; W+FR, continuous white light supplemented with far-red light. Five-day-old seedlings grown under either W or W+FR condition were treated with MOCK, NPA, or PAC for 5 days on 1/2 MS media. Scare bar, 5 mm. **(B)** Hypocotyl lengths were measured at the indicated times: 0 day, 1 day, 3 days, and 5 days after NPA or PAC treatment. Data shown are mean ± SD (n > 15). **(C–E)** Expression of *GA20ox1* **(C)**, *GA20ox2* **(D)**, and *GA3ox1* **(E)** under W and W+FR in time course using 7-day-old seedlings were analyzed by real-time quantitative PCR. Results are representative of three independent experiments. Data shown are mean ± SD. All data were statistically evaluated using one-way ANOVA: *P < 0.05; **P < 0.01; ***P < 0.005.

To obtain an insight into a relative contribution of GA in mediating shade response, hypocotyl elongation of Arabidopsis seedlings grown under shade were monitored after treating with inhibitors of auxin and GA metabolism. Reflecting the well-established role of auxin during the onset and the early stage of the shade avoidance response (Casal, 2013; Wang et al., 2020), seedlings treated with an auxin transport inhibitor, naphthylphthalamic acid (NPA) (Abas et al., 2020), failed to exhibit the characteristic hypocotyl elongation under shade as expected. Similarly, the seedlings treated with an inhibitor of GA biosynthesis, paclobutrazol (PAC/PBZ) (Rademacher, 2000), also showed a noticeably reduced hypocotyl length under shade, indicating that GA also plays a role in mediating the shade response as much as auxin does (**Figures 1A, B**).

Consistent with this observation, expressions of *GA20ox1*, *GA20ox2*, and *GA3ox1*, three key regulators of GA biosynthesis in *Arabidopsis* (Hedden et al., 2001; Rieu et al., 2008), were found to be induced over a 4-hr period of shade treatment (**Figures 1C–E**). GA20 oxidase (GA20ox) mediates several conversion steps in the late stage of GA biosynthetic pathway occurring in the cytoplasm and GA3 oxidase (GA3ox) catalyzes the final step of producing biologically active form of GA, GA1 and GA4 (Hedden, 2020). Of the two major isoforms of GA20 oxidase in *Arabidopsis*, our result revealed that the induction of *GA20ox1* expression preceded that of *GA20ox2*, reaching at the peak within an hour of shade treatment, which was followed by the induction of *GA20ox2* expression within next couple of hours.

### 3.2 ATHB2 acts as a positive regulator of *GA20ox2* expression

**Figure 2:**
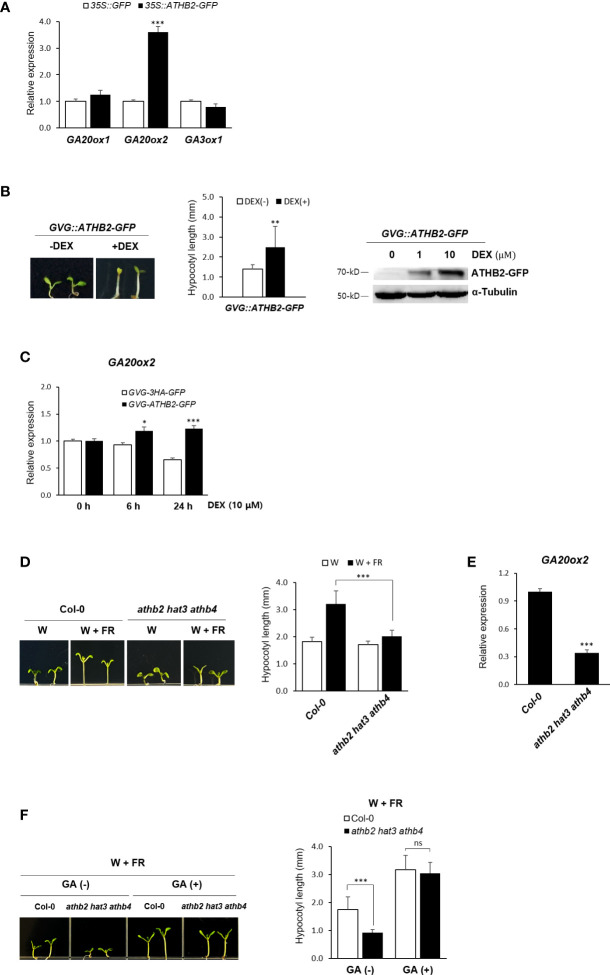
Regulation of *GA20ox2* expression in *ATHB2*-expressing protoplasts and transgenic plants. **(A)** Expression of *GA20ox1*, *GA20ox2*, and *GA2ox3* was analyzed in *Arabidopsis* protoplasts transfected with plasmids containing *35S::ATHB2-GFP* or *35S::GFP* by real-time quantitative PCR. **(B)** Expression of *ATHB2* in transgenic plants with *GVG::ATHB2-GFP* was examined after treatment with dexamethasone (DEX). Seven-day-old seedlings were grown on 1/2 MS media treated with 0.01% ethanol (-DEX) or 10 μM DEX (+DEX) (left) and their hypocotyl lengths were measured (center). Data shown are mean ± SD (n > 15). Western blot analysis using 7-day-old plants treated with DEX was performed with antisera against GFP of α tubulin (right). **(C)** *GA20ox2* expression was examined in transgenic plants with *GVG::ATHB2-GFP* treated with DEX for 0 h, 6 h, and 24 h by real-time quantitative PCR. Results are representative of three independent experiments. Data shown are mean ± SD (n > 3). **(D)** A triple-knockout mutant of *ATHB2* paralogues (*athb2 hat3 athb4*) was grown under W or W+FR conditions and their hypocotyl lengths were measured. Data shown are mean ± SD (n > 20). **(E)** *GA20ox2* expression was examined in 7-day-old seedlings of *athb2 hat3 athb4*. Results are representative of three independent experiments. **(F)** A triple-knockout mutant of *ATHB2* paralogues (*athb2 hat3 athb4*) was grown under W+FR condition in the absence/presence of GA and their hypocotyl lengths were measured. Data shown are mean ± SD (n > 20). Data shown are mean ± SD (n > 3). All data were statistically evaluated using one-way ANOVA: *P < 0.05; **P < 0.01; ***P < 0.005. ns, no significant difference.

The rapid transcriptional activation of the GA biosynthetic genes under shade condition observed in our result was reminiscent of the quick induction kinetics of *ATHB2* during the onset of the shade avoidance response (Carabelli et al., 1996; Steindler et al., 1999; Carabelli et al., 2013). We therefore tested if any of these genes’ expression is regulated by ATHB2 as part of the shade avoidance response. Protoplasts derived from Arabidopsis leaf mesophyll cells were transfected with a plasmid construct expressing the *ATHB2* and the transcript levels of *GA20ox1*, *GA20ox2*, and *GA3ox1* were measured by real-time quantitative PCR analysis. To a little surprise, the ectopic expression of *ATHB2* resulted in an increased expression of the endogenous *GA20ox2* only, while those of *GA20ox1* and *GA3ox1* remain largely unchanged (**Figure 2A**), indicating that the regulatory effect of ATHB2 is limited to the control of *GA20ox2* expression despite all these three genes were induced under shade condition.

The observed transcriptional activation of *GA20ox2* by ATHB2 was also confirmed in transgenic Arabidopsis plants expressing a dexamethasone (DEX)-inducible *ATHB2-GFP* fusion construct as well as in a triple-knockout mutant of *ATHB2* paralogues (*athb2 hat3 athb4*). Upon DEX treatment, the transgenic plants displayed a characteristic hypocotyl elongation phenotype of the shade response even under light condition, demonstrating a central role of ATHB2 in maintaining the shade avoidance response (**Figure 2B**). Accordingly, expression of *GA20ox2* was found to be increased along with the ATHB2 induction in response to the DEX treatment in the transgenic plants (**Figures 2C**, **S1**). In addition, the hypocotyl elongation phenotype in DEX-treated transgenic plants expressing *ATHB2-GFP* was not visible with PAC treatment (**Supplementary Figure S2**). On the other hand, noticeably reduced hypocotyl elongation under shade and the *GA20ox2* expression was observed with the triple mutant of *ATHB2* paralogues (*athb2 hat3 athb4*) (**Figures 2D, E**). However, the hypocotyl elongation of the triple mutant plants under shade condition was equivalent to that of the wild-type seedlings when they were provided with exogenous GA treatment (**Figure 2F**), revealing that the observed mutant phenotype was mainly due to a defect in GA biosynthesis. While there might be other unidentified mechanism by which GA biosynthesis is regulated and under the control of the ATHB2, at least our results may indicate that one of the roles of ATHB2 in coordinating the shade response is to stimulate GA biosynthesis through the transcriptional activation of *GA20ox2*, thereby contributing to the hypocotyl elongation.

### 3.3 ATHB2 transactivates *GA20ox2* by directly binding to a unique *cis* element on the promoter

**Figure 3:**
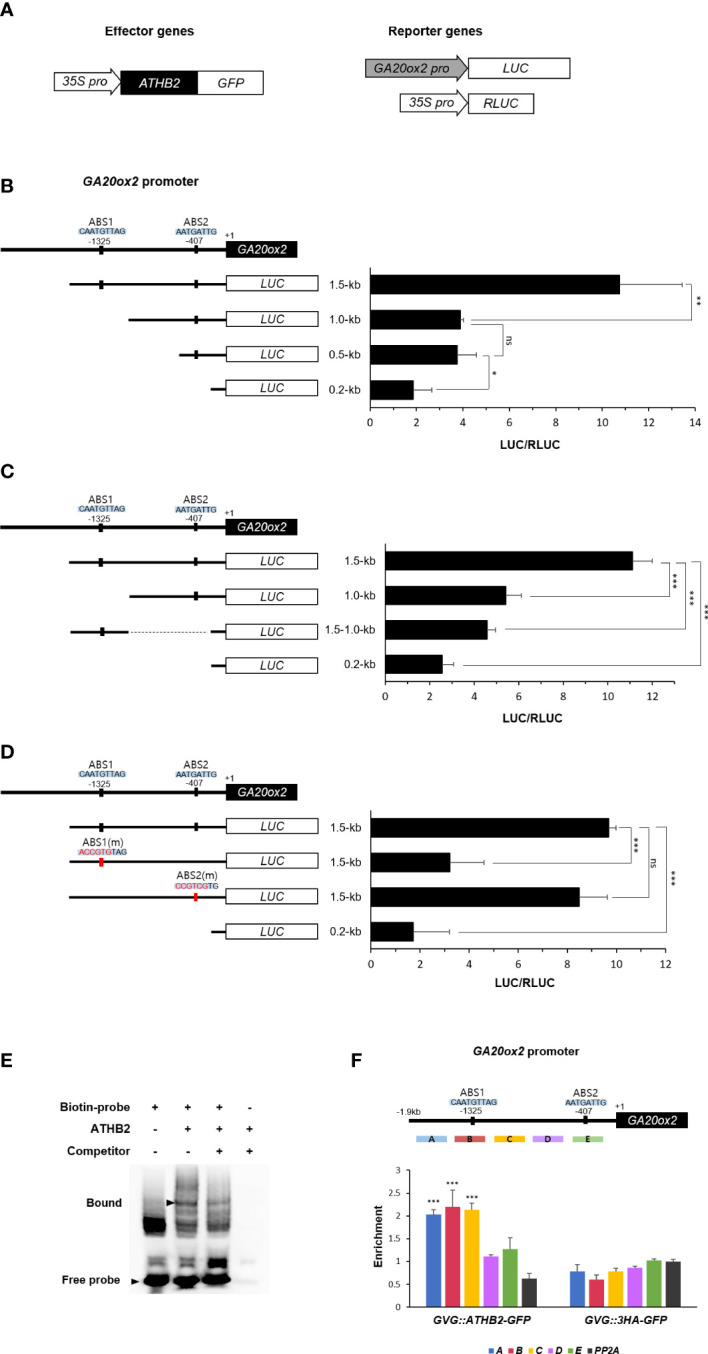
Activation of *GA20ox2* by ATHB2 binding to an upstream region of *GA20ox2*. **(A)** Luciferase assays using different lengths of *GA20ox2* upstream region fused with firefly luciferase (*GA20ox2 pro::LUC*) as reporters and *35S pro::ATHB2-GFP* as an effector construct were performed. **(B)** Different lengths of *GA20ox2* upstream region (1.5-kb, 1.0-kb, 0.5-kb, and 0.2-kb upstream regions) fused with firefly luciferase were used as reporter constructs, and *35S pro::RLUC* was used as internal control. Firefly luciferase (LUC) activity was normalized with *Renilla* luciferase (RLUC) activity. The diagram shows two putative ATHB2 binding sites (ABS1 and ABS2) in *GA20ox2* upstream region. **(C)** A region of the *GA20ox2* promoter occupying between -1.0-kb ~ -1.5-kb upstream was fused to a *LUC* reporter with the minimal promoter region of *GA20ox2* (-0.2 kb fragment) tested for the activation activity. **(D)** ABS1 and ABS2 on *GA20ox2* promoter were mutated, respectively; ABS1(m) and ABS2(m). The resulting constructs were used for luciferase assay. **(E)** An electrophoretic mobility shift assay (EMSA) was performed using a 31-mer of biotin-labeled oligonucleotides flanking the ABS1 sequence. Purified 6X His-tagged ATHB2 protein was incubated with labelled oligonucleotides (second lane). The same EMSA experiment with 100X molar excess of unlabeled oligonucleotides as competitor was examined as well for indicating the specificity of ATHB2 binding to ABS1. Arrowheads indicate free and bound oligonucleotides separated in a native gel. **(F)** ChIP assay showing enrichments of *GA20ox2* promoter fragments by ATHB2. Schematic diagram indicates ATHB binding sites (ABS1 and ABS2) and ChIP amplicons **(A-E)** in the upstream region of *GA20ox2*. The enrichment of each DNA fragment was normalized by the level of input DNA and the ChIP enrichment value of the control *PP2A* promoter. Asterisks indicate significant differences from the enrichment of *PP2A* genomic fragments Results are representative of three independent experiments. Data shown are mean ± SD (n=3). All data were statistically evaluated using one-way ANOVA: *P < 0.05; **P < 0.01; ***P < 0.005; ns, no significant difference.

It has been reported that the transcription of *GA20ox2* in *Arabidopsis* is mainly regulated through a GA-feedback mechanism mediated by DELLA-GAF1 complex, in which GAF1 recruits DELLA that functions as a GA concentration-dependent transcriptional co-activator in this occasion (Fukazawa et al., 2014; Fukazawa et al., 2017). As multiple GAF1-binding sites have been identified on the *GA20ox2* promoter (Fukazawa et al., 2017), it is possible that the positive effect of ATHB2 on the *GA20ox* expression could be mediated as a component of the DELLA-GAF1 complex. Thus, we tried to locate approximate site of ATHB2 action on *GA20ox2* promoter by comparing relative transcriptional activity of several deletion versions of the *GA20ox2* promoter fragments driving the luciferase reporter construct after transient expression in *Arabidopsis* protoplasts. The result showed that a region of the *GA20ox2* promoter occupying between -1,000 ~ -1,500 bp upstream was specifically responsible for the ATHB2-dependent transcriptional activation of *GA20ox2* (**Figures 3A, B**). As this region is outside of the area where all the GAF1-binding elements (*cisA* – *cisE*) have been located (Fukazawa et al., 2017), the stimulatory effect of ATHB2 may operates independently of that of well-established DELLA-GAF1 transactivating complex. Thus, in order to determine if this region alone was sufficient for conferring the stimulatory effect of ATHB2 on the *GA20ox2* promoter, this fragment was directly fused to a *LUC* reporter with the minimal promoter region of the *GA20ox2* (-0.2 kb fragment) and tested for its transcriptional activity along with other partial fragments of *GA20ox2* promoter-reporter constructs (**Figure 3C**). Based upon the result, it appears that although the region is necessary for ATHB2 to implement the stimulatory effect on the *GA20ox2* expression, but not sufficient for exerting the effect alone, suggesting that it may require additional positive regulator(s) acting on other regions of the promoter for an optimal activity.

A putative binding site for plant HD-zip domain transcription factors (Sessa et al., 1993) was identified within the region (CAATGTTAG), which we tentatively named ABS1, and altering its sequence into ACCGTGTAG abolished the ATHB2-dependent transcriptional activation of the reporter construct conferred by the fragment. On the other hand, a mutation of a similar sequence found at more proximal end of the promoter (-407 from the transcription start site), which also contains a conserved core sequence of the HD-zip domain binding motif (named ABS2), did not affect the reporter gene expression, suggesting that the observed effect is likely to be mediated through the ABS1 sequence element (**Figure 3D**).

To test if ATHB2 can directly bind to the ABS1 sequence motif, we carried out an electrophoretic mobility shift assay (EMSA) using biotin-labeled oligonucleotides of the ABS1 sequence and recombinant ATHB2 protein expressed in and purified from *E. coli*. As shown in **Figure 3E**, an ATHB2-specific electrophoretic mobility shift of ABS1-containing oligonucleotides was detected, demonstrating that ATHB2 can directly bind to the DNA having the ABS1 sequence motif (GAATGTTAG) at least *in vitro*. This was further verified *in vivo* by chromatin immunoprecipitation (ChIP) – PCR analysis with the DNA samples isolated from transgenic Arabidopsis expressing *ATHB2-GFP*, the result of which showed the ABS1-containing area of the *GA20ox2* promoter as the most strongly amplified region (**Figure 3F**).

### 3.4 *GA20ox2* expression is negatively regulated by TCP13

**Figure 4:**
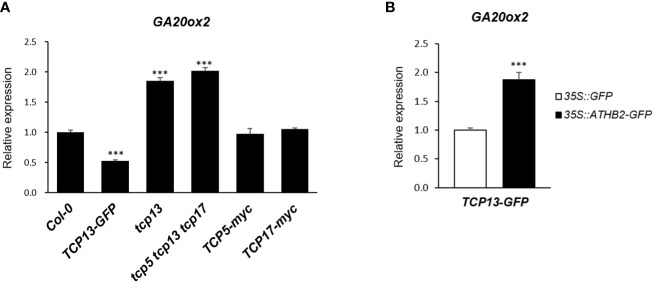
Regulation of *GA20ox2* expression in *TCP13*-expressing transgenic plants, protoplasts, and mutants. **(A)** Expression of *GA20ox2* in *Arabidopsis* seedlings with 35S promoter-driven *TCP13-GFP* fusion construct (*TCP13-GFP*), tcp13 mutant, triple KO mutant for *TCP13* and its paralogs, *TCP5* and *TCP17* (*tcp5 tcp13 tcp17*), seedlings with 35S promoter-driven *TCP5-myc* fusion construct (*TCP5-myc*), and those with 35S promoter-driven *TCP17-myc* fusion construct (*TCP17-myc*) examined by real-time quantitative PCR. **(B)** Expression of *GA20ox2* in *Arabidopsis* protoplasts of *TCP13-GFP* transgenic plants that were subsequently transfected with 35S promoter-driven *ATHB2-GFP* fusion construct (*35S pro::ATHB2-GFP*) examined by real-time quantitative PCR. More than 3 independent transgenic plants expressing *TCP5*, *TCP13* or *TCP17*, respectively. Results are representative of more than three independent experiments. Data shown are mean ± SD (n > 3). All data were statistically evaluated using one-way ANOVA: ***P < 0.005.

Another key regulator of shade-specific gene expression we have followed for years is TCP13, a member of plant-specific transcription factor with a unique helix-loop-helix motif (Aggarwal et al., 2010). TCP13 and its close paralogues of the CIN-like TCPs, TCP5 and TCP17, have been implicated in a number of developmental and physiological processes of plants including shade avoidance response, thermomorphogenesis, GA biosynthesis and cell expansion, many of which are accomplished through functional association with other regulators such as PIFs (Zhou et al., 2018; Han et al., 2019; Zhou et al., 2019; Rath et al., 2022). Therefore, we considered a possibility of ATHB2 regulating the *GA20ox2* transcription as a functional complex with TCP13 and examined if there was any additive effect of TCP13 on the expression of *GA20ox2* together with ATHB2. On the contrary to what we have anticipated, however, *GA20ox2* transcript level was found to be significantly lower in our transgenic Arabidopsis constitutively expressing *TCP13-GFP* construct, which was also corroborated in triple mutant of *tcp5 tcp13 tcp17* that showed significantly elevated level of the *GA20ox2* expression (**Figure 4A**). Of particular interest regarding the relative roles of these TCP13 paralogues in the regulation of *GA20ox2* expression uncovered from this result was that only TCP13, but not TCP5 or TCP17, appears to be acting as a repressor of *GA20ox2* expression, as evidenced in the result of the transgenic plants expressing *TCP5* (*TCP5-myc*) or *TCP17* (*TCP17-myc*) as well as in the result of the *TCP13* knockout mutant (*tcp13*) in comparison with the triple mutant (**Figure 4A**). We attempted to confirm this negative role of TCP13 in a transient assay of protoplast transfection. Although ectopic expressions of both *TCP13* and *ATHB2* were examined (**Figure S3**), they may not show their respective effects on *GA20ox2* expression under the real physiological condition, considering their expression pattern. Thus, we tried to observe the effect of TCP13 on the *GA20ox2* expression in an *ATHB2*-expressing background since the two proteins have shown a specific interaction in yeast two-hybrid analysis (Hur et al., 2019), thus it could potentially result in a confounding effect. When protoplasts made from the *TCP13-GFP* transgenic plants were transfected with a plasmid expressing *ATHB2* and assayed for the expression of endogenous *GA20ox2*, derepression of *GA20ox2* transcription by the ectopic expression of the *ATHB2* gene was evident, indicating an antagonistic regulatory effect of the two on the regulation of *GA20ox2* expression (**Figure 4B**).

### 3.5 The site of repression by TCP13 on *GA20ox2* promoter does not overlap with the ATHB2-specific element

**Figure 5:**
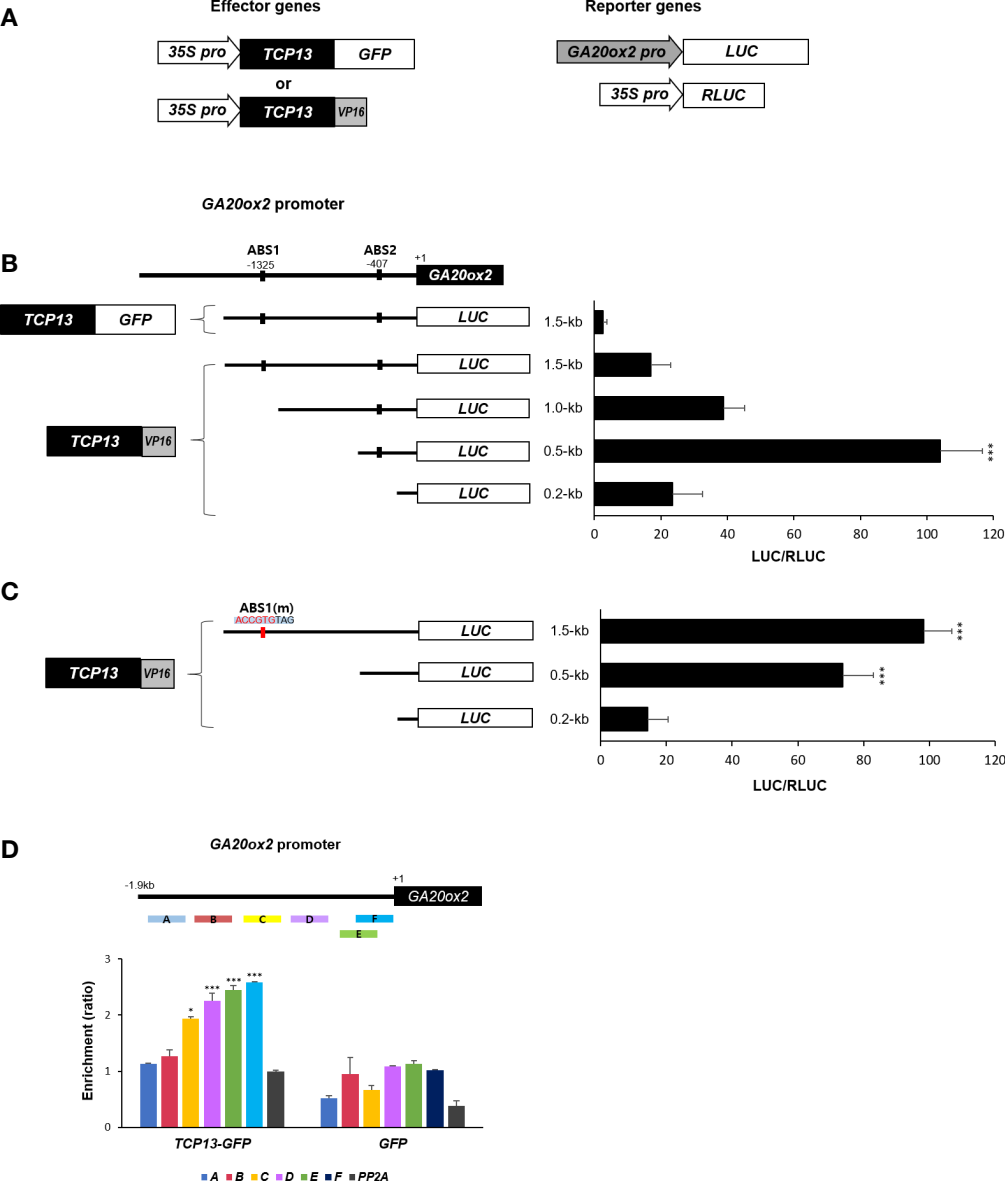
Regulation of *GA20ox2* by TCP13. **(A)** Luciferase assays using different lengths of *GA20ox2* upstream region fused with firefly luciferase (*GA20ox2 pro::LUC*) as reporters and *35S pro::TCP13-GFP* or *35S pro::TCP13-VP16* as effector constructs were performed. **(B)** Different lengths of *GA20ox2* upstream region (1.5-kb, 1.0-kb, 0.5-kb, and 0.2-kb upstream regions) fused with firefly luciferase were used as reporter constructs, and *35S pro::RLUC* was used as internal control. Firefly luciferase (LUC) activity was normalized with *Renilla* luciferase (RLUC) activity. **(C)** ABS1 on *GA20ox2* promoter was mutated; ABS1(m). The resulting construct was used for luciferase assay. **(D)** ChIP assay showing enrichments of *GA20ox2* promoter fragments by TCP13. Schematic diagram indicates ChIP amplicons **(A–F)** in the upstream region of *GA20ox2*. The enrichment of each DNA fragment was normalized by the level of input DNA and the ChIP enrichment value of the control *PP2A* promoter. Asterisks indicate significant differences from the enrichment of *PP2A* genomic fragments. Results are representative of three independent experiments. Data shown are mean ± SD (n=3). All data were statistically evaluated using one-way ANOVA: *P < 0.05; ***P < 0.005.

It was intriguing to contemplate the mechanism of transcriptional repression of *GA20ox2* brought about by TCP13. For instance, if it requires formation of heterodimer with ATHB2 it is conceivable that the inhibitory effect of TCP13 is mainly achieved through titrating the ATHB2 out of the *GA20ox2* promoter. Therefore, we attempted to determine whether TCP13 indeed binds to the promoter of *GA20ox2*, and if so, the binding region coincides with the area where the ABS1 sequence is present. A chimeric construct having the activation domain of VP16 (Herpes Simplex Virion Protein 16) fused to TCP13 ORF at its C-terminus was generated and *Arabidopsis* protoplasts were transfected with this construct together with the plasmid expressing the luciferase under *GA20ox2* promoter (**Figure 5A**). Truncated versions of the *GA20ox2* promoter fragments were used to examine the presence of any region that displays a TCP13-dependent (TCP13-VP16) transactivation of the reporter. The result revealed that a promoter fragment spanning to - 500 bp upstream of the transcription start site was the only one conferred a significant activation of the reporter gene expression (0.5-kb fragment in the **Figure 5B**). The originally observed repressive function of the TCP13 on the *GA20ox2* expression was also evident in this assay, as shown with the TCP13 construct fused with GFP instead of the VP16 cotransfected with the reporter driven by the full-length 1.5-kb promoter (**Figure 5B**). With this result, therefore, it could be concluded that TCP13 acts as a repressor of *GA20ox2* binding to a specific promoter region which does not overlap with the area containing the putative ATHB2 binding site, ABS1.

One puzzling finding out of this experiment, however, was that the full-length promoter did not show as much transcriptional activation as with the 0.5-kb promoter fragment which was still included in the full-length version of the promoter. We speculated that there might be a sequence-specific protein factor interfering the operation of TCP13 elsewhere on the promoter region of the *GA20ox2*. A possible candidate for such interference could be the formation of heterodimer between the endogenous ATHB2 and the ectopically expressed TCP13-VP16 proteins being recruited onto the ABS1 sequence on the *GA20ox2* promoter. To test this possibility, we repeated the same transfection assay with the *GA20ox2* promoter having the ABS1 sequence (CAATGTTAG) changed into ABS1(m) (ACCGTGTAG), which abolished the stimulatory effect of ATHB2 on *GA20ox2* expression in the result summarized in the **Figure 3C**. We thought that by abolishing possible influence of the endogenous ATHB2 by eliminating its binding site on the promoter of the reporter gene, the stimulatory effect of TCP13-VP16 on the full-length promoter would be restored. In support of our hypothesis, a dramatic increase in the activation of the reporter gene expression was observed with the mutant full-length promoter of *GA20ox2*, even higher than with that of the 0.5-kb fragment of the wild-type promoter (**Figure 5C**). To clearly test if TCP13 can directly bind to the 0.5-kb fragment of *GA20ox2* promoter, we performed chromatin immunoprecipitation (ChIP) – PCR analysis with the DNA samples isolated from transgenic Arabidopsis expressing *TCP13-GFP*. Result showed possibly the TCP13 binding to the 0.5-kb fragment of *GA20ox2* promoter (**Figure 5D**) although the binding region needs further specification. Therefore, it appears that ATHB2 and TCP13 are acting antagonistically in regulating the *GA20ox2* expression, playing a stimulatory and a repressive role, respectively.

### 3.6 TCP13 can specifically interact with GAF1

**Figure 6:**
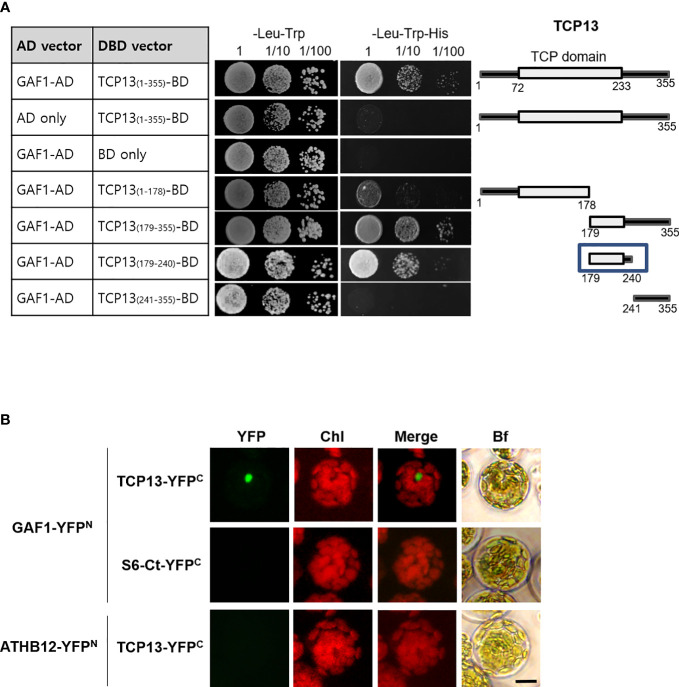
Interaction of TCP13 with GAF1. **(A)** Different regions of TCP13 were tested for the interaction with GAF1 in yeast two-hybrid assay. The DNA-binding domain (DBD) of GAL4 was fused to different regions of TCP13 while the activation domain (AD) of GAL4 was fused to GAF1. Co-transformed yeasts were selected on media lacking Leu and Trp (-Leu-Trp), and selected on media lacking Leu and Trp and His (-Leu-Trp-His) with or without 1 mM 3-amino-1,2,4 triazole (3-AT). The minimal region of TCP13 required for the interaction with GAF1 was indicated as a box. **(B)** Bimolecular fluorescence complementation (BiFC) analysis of interaction between TCP13 and GAF1. *Arabidopsis* protoplasts were co-transfected with several combinations of the constructs of *P_35S_::GAF1-YFP^N^* and *P_35S_::TCP13-YFP^C^*. S6-Ct-YFP^C^: truncated RPS6 fused to YFP. A transcription factor ATHB12 fused to the N-terminal fragment of YFP (ATHB12-YFP^N^) was used as negative control. Bf, bright field. BiFC experiments were replicated three times with similar results. Scale bar = 10 μm.

Since the GAF1-DELLA *trans*-activator complex has been reported as the principal regulator for the expression of *GA20ox2* in *Arabidopsis* (Fukazawa et al., 2017), the inhibitory role of TCP13 on *GA20ox2* disclosed in our present study made us wonder if this activity might involve a physical interaction between TCP13 and GAF1. In line with the presence of the intrinsically disordered region (IDR) at the C-terminus (Dunker et al., 2001), a characteristic feature of all TCP proteins that is thought to be responsible for establishing interactions with multiple different proteins by providing flexible structural platform (Valsecchi et al., 2013), a number of proteins have been identified as capable of forming a specific interaction with TCP13 (http://interactome.dfci.harvard.edu, http://zzdlab.com/plappisite). As GAF1 is not included in the list of these interacting proteins, however, we inquired a possible interaction between GAF1 and TCP13 by conducting yeast two-hybrid analyses, in which several partial fragments as well as the full-length TCP13 protein were probed with the full-length GAF1 protein for the interaction. The result indicated that TCP13 can interact with GAF1 and the region between the amino acid residues 179 and 240, which includes the C-terminal portion of the TCP domain and a few more amino acids beyond, were found to be responsible for the interaction (**Figure 6A**). This interaction between TCP13 and GAF1 was also confirmed *in planta* by bimolecular fluorescence complementation (BiFC) analysis, in which full-length fragments of GAF1 and TCP13 were fused to N-terminal and C-terminal halves of YFP, respectively, and demonstrated their specific interaction (**Figure 6B**). Therefore, it seems probable that the repression of *GA20ox2* by TCP13 may be achieved through inhibition of GAF1-dependent transactivation of the gene, by interfering the formation of an active DELLA-GAF1 complex. However, one caveat of this idea is that the site of TCP13 action on the *GA20ox2* promoter, as we have determined by the results of **Figure 5**, does not overlap with any of the GAF1-binding sites identified on the *GA20ox2* promoter, *cis* A through E (Fukazawa et al., 2017).

## 4 Discussion

Our present study uncovered the role of ATHB2 as a positive regulator of GA biosynthesis by transactivating the expression of *GA20ox2*. Thus, as a downstream target of the PIF4/PIF5 activity, ATHB2 appears to coordinate the synthesis of GA which is an essential component of the early-stage shade avoidance response. While it is not clear whether the induction of ATHB2 is a result of direct activation by PIF4/PIF5 during the procedure or the one requiring the auxin biosynthesis as a prerequisite as was recently proposed (Küpers et al., 2023), ATHB2 seems to play a key role in bridging the auxin and GA signaling together to facilitate the shade avoidance response.

### 4.1 ATHB2 activates *GA20ox2* expression *via* a specific sequence element

Our result identified a *cis*-regulatory sequence element specifically associated with the stimulatory effect of ATHB2 on *GA20ox2* promoter. The sequence motif, which we tentatively named ABS1, is highly homologous to the consensus sequence motif known for the plant HD- domain transcription factors (Sessa et al., 1993). Presence of the ABS1 appears to be essential for the ATHB2-dependent *trans*activation of the *GA20ox2* gene, as revealed from our promoter deletion analysis together with the effect of mutating its sequence on the reporter gene expression. Moreover, direct binding of ATHB2 to ABS1 sequence was shown by EMSA and ChIP assay (**Figures 3D, E**), thus ATHB2 is likely to act directly on the *GA20ox2* promoter through ABS1 sequence in regulating its expression in response to the onset of shade condition.

Even though both the *GA20ox1* and *GA20ox2* are induced under shade, our result showed that only the *GA20ox2* expression was regulated by ATHB2, which is in consistence with that no ABS1 sequence motif is found on the promoter of *GA20ox1*. By contrast, both genes have been reported to be under the positive control by the DELLA-GAF1 complex, which incorporates the GA-feedback regulation of these genes (Fukazawa et al., 2017; Fukazawa et al., 2021). Therefore, while it has not been determined if the GAF1/DELLA regulatory machinery is also in operation under the shade condition, the shade-specific expression of *GA20ox2* seems to have obtained a unique element of control from ATHB2 and thereby delineates from the control of *GA20ox1* expression under the same condition.

### 4.2 TCP13 plays as a repressor of *GA20ox2* and acts independently of ATHB2

As ATHB2 has been identified to be interacting with TCP13 (Hur et al., 2019), a member of a plant-specific transcription factor family having noncanonical bHLH domain (Martin-Trillo and Cubas, 2010), we contemplated possible formation of a functional heterodimer between ATHB2 and TCP13 in transactivating the *GA20ox2* expression. However, our results showed that TCP13 functioned as a repressor of *GA20ox2*, casting a scrutiny regarding the biological relevance and possible mechanism of the observed repression brought up by TCP13. The *GA20ox2*-specific repressor activity of TCP13 was initially detected in the protoplast transfection experiment and was also confirmed in transgenic plants over-expressing *TCP13-GFP* construct as well as in the single (*tcp13*) and triple knock-out mutant (*tcp5 tcp13 tcp17*), validating its biological relevance.

The question remained as to whether the repressor function of TCP13 involved in the change in the ATHB2 activity, reducing its stimulatory effect by titrating it out of the *GA20ox2* promoter, for example. In such a scenario, the site of TCP13 action on the promoter would be mapped to be overlapping with that of ATHB2. Thus, to map the site of TCP13 activity on the *GA20ox2* promoter we designed a chimeric construct having the VP16 transcription activation domain fused to the C-terminus of TCP13 and used it as an effector in protoplast transfection assays with deletion fragments of *GA20ox2* promoter driving the reporter luciferase expression (**Figure 5A**). The result indicated that TCP13 exercises its effect on a location of the *GA20ox2* promoter different from the area where the ATHB2 plays its stimulatory action through its putative binding site, ABS1 (**Figure 5B**).

### 4.3 TCP13 appears to act on the promoter *GA20ox2* through direct binding

An additional important understatement of this result is that TCP13 appears to bind directly to a promoter region of *GA20ox2*. In contrast to the original inhibitory activity of TCP13 on the *GA20ox2* expression, our chimeric construct was designed to confer a stimulatory effect through the fused VP16 activation domain. If TCP13 did not directly bind to the promoter of *GA20ox2*, no positive effect of VP16 could have been possibly detected in our reporter assays in this scheme. A putative TCP13-specific element presumably serving as a direct binding site for the TCP13 may be present within the 0.5 kb-promoter fragment most proximal to the TCP13 ORF based on our result. The consensus sequence motif for binding of the TCP-family transcription factors has been known as GGNCCCAG for the class I TCPs and G(G/T)GGNCC(A/C) for the class II TCPs (Kosugi and Ohashi, 2002; Aggarwal et al., 2010), to which the TCP13 belongs. Although no sequences exactly matching to this consensus motif were found within the 0.5-kb promoter fragment, a sequence similar to the consensus, GTTGGCCA, was present within the fragment at about 400 bp upstream of a putative TATA element. Further detailed analyses on this promoter region, with respect to both the *cis*-elements and protein factors associated with them would be able to provide more comprehensive insight into the mechanism of the transcriptional repression of *GA20ox2* mediated by TCP13.

One highly speculative but potentially interesting scenario is a possibility of TCP13 playing an inhibitory role against the DELLA-GAF1 transactivation complex. Although there is no concrete evidence for the DELLA-GAF1 in operation to control *GA20ox* gene expression under shade, the induction kinetics of *GA20ox1* and *GA20ox2* under shade condition appears to be reminiscent of the GA feedback inhibition. As the activity of DELLA-GAF1 complex is known to be repressed by a transcriptional corepressor TPR (TOPLESS-Related) (Fukazawa et al., 2014) and it has been also reported that TCP13 can specifically interact with TPR in an earlier study (Tao et al., 2013), it may be possible for TCP13 to participate in this regulatory complex as the one mediating the recruitment of the repressive factor of TPR to the promoter of *GA20ox2*. In support of this idea, we detected a specific interaction between TCP13 and GAF1 in our yeast two-hybrid and BiFC assays (**Figure 6**). However, at this point this idea of interdependency of ATHB2 and TCP13 to the GAF1 function has not been supported by any experimental evidence and can only be further tested and evaluated in later studies involving *gaf1* mutant plants and necessary rigorous molecular analyses.

### 4.4 Possible role of ATHB2 in regulating *GA20ox2* expression with respect to TCP13

One peculiar result obtained from the promoter deletion experiment with our TCP13- VP16 chimeric construct was that the luciferase activity obtained from the full-length promoter was significantly lower than that of the smaller, - 0.5-kb fragment which contained the putative TCP13 binding sequence. A straightforward account for this observation would be a presence of an inhibitory activity for the function of the TCP13 outside of the - 0.5-kb promoter region. One immediate suspect for this activity that came to our mind was ATHB2, as it is capable of having a specific interaction with TCP13. Having the transfection assay with the chimeric TCP13-VP16 construct with ABS1(m), the mutated ABS1 sequence, in the *GA20ox2* promoter, we were able to demonstrate that ABS1 sequence was responsible for suppressing the activity of TCP13, suggesting that ATHB2, either directly or indirectly through other unknown protein factor(s), plays as a derepressor for the expression of *GA20ox2* under shade response in *Arabidopsis*. TCP13 may bind to 0.5-kb region of the *GA20ox2* promoter and then as *ATHB2* is rapidly induced upon shade treatment, such functional relation between ATHB2 and TCP13 would allow the induction of *GA20ox2* expression in response to the onset of the shade condition. Therefore, the role of ATHB2 in the regulation of *GA20ox2* expression may be in a shade-specific lifting of the inhibitory effect of TCP13 to enable the stimulatory role of GAF1-DELLA complex to initiate the early-stage activation of *GA20ox2*, followed by GA-dependent feedback inhibition (Fukazawa et al., 2017). Further studies regarding molecular details of the interaction between TCP13 and GAF1 and investigation of its biological significance, including possible involvement of GAF1 in the shade avoidance response as well, should be able to provide much clearer understanding of complex regulatory networks coordinating the adaptive response of plant under shade condition.

## Data availability statement

The raw data supporting the conclusions of this article will be made available by the authors, without undue reservation.

## Author contributions

OS and CZ designed the experiments and made and analyzed transgenic plants; XY, LD, and Y-SH performed assays and yeast works; CZ, KN, and S-YC analyzed the transgenic plants and interpreted the data. C-IC and SK coordinated the study and finalized the article. All authors contributed to the article and approved the submitted version.
