# Supplementary material for: Identification of GA20ox2 as a target of ATHB2 and TCP13 during shade response
Source: Front Plant Sci. 2023 Apr 21;14:1158288. doi: 10.3389/fpls.2023.1158288 (PMC10160606; doi:10.3389/fpls.2023.1158288)
Supplement: Supplementary file 1 [file DataSheet_1.pdf]

Contribution to the field statement

This is a tale of two transcription factors, a beginning chapter. One has been known as a key initiator of an adaptive response of plant under shade known as the "shade avoidance syndrome (SAS)" but its exact target during the response has been elusive. The other has been implicated in many important physiological and developmental processes and its role in the SAS is also emerging more recently. We have long suspected these two proteins might work together in the SAS response, but we did not expect they would act antagonistically as revealed by our work here. They are members of two distinct groups of plant-specific transcription factors, respectively, functions of whose members are involved in many vital plant physiologies. Our present work reports an unlikely partnership between these two group's members and invites more questions regarding the molecular mechanism by which they play their regulatory roles on the expression of a gene required for the onset of the SAS response. Since this gene has been known as the target of another renowned transcription factor, our work would also warrant further investigation on the functional and molecular relationship among these three proteins in the regulation of this gene's expression.
