# Supplementary material for: Identification of GA20ox2 as a target of ATHB2 and TCP13 during shade response
Source: Front Plant Sci. 2023 Apr 21;14:1158288. doi: 10.3389/fpls.2023.1158288 (PMC10160606; doi:10.3389/fpls.2023.1158288)
Supplement: Supplementary file 2 [file DataSheet_2.pdf]

Supplementary figures

Son et al., Figure S1.

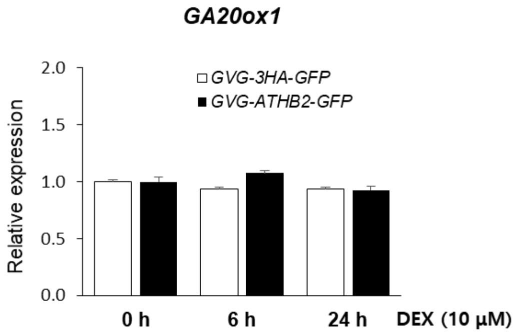

Figure S1. Expression of *GA20ox1* in DEX-treated plants with *GVG::ATHB2-GFP*. *GA20ox1* expression was examined in transgenic plants with *GVG::ATHB2-GFP* or *GVG::3HA-GFP* after treatment with DEX for 0 h, 6 h, and 24 h by real-time quantitative PCR. Results are representative of three independent experiments. Data shown are mean ± SD (n > 3).

Son et al., Figure S2.

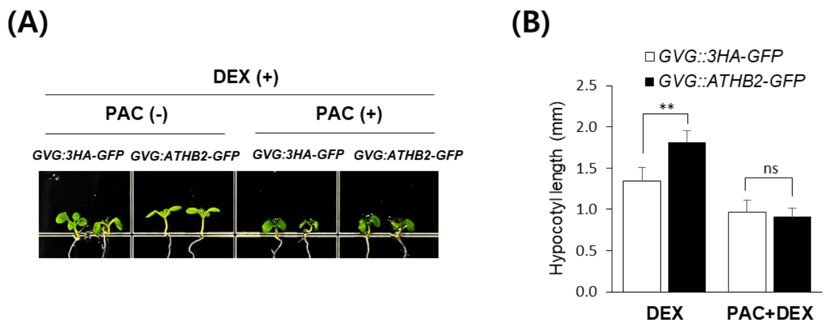

Figure S2. (A) Effects of GA biosynthetic inhibitor PAC on hypocotyl lengths of DEX-treated plants with *GVG::ATHB2-GFP* or *GVG::3HA-GFP*. Five-day-old seedlings were grown on 1/2 MS media containing 10 μM DEX alone (DEX+, PAC-), or DEX and PAC (DEX+, PAC+). (B) Their hypocotyl lengths were measured at the same stage. Data shown are mean ± SD (n > 15). Results are representative of three independent experiments. All data were statistically evaluated using one-way ANOVA: **, 0.005< P <0.01 ; ns, no significant difference.

Son et al., Figure S3.

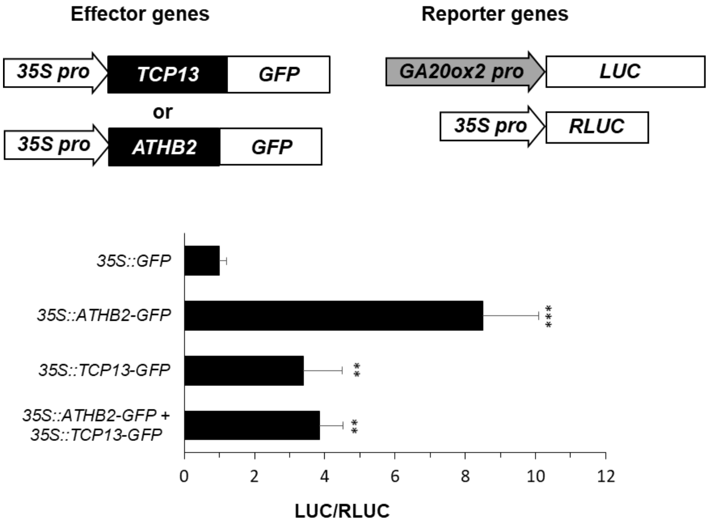

Figure S3. Expression of *GA20ox2* by ATHB2 and TCP13. Luciferase assays using 1.5-kb lengths of *GA20ox2* upstream region fused to firefly luciferase (*GA20ox2 pro::LUC*) as reporters, and *35S pro::ATHB2-GFP* and *35S pro::TCP13-GFP* constructs as effectors were performed. A construct of *35S pro::RLUC* was used as internal control. Firefly luciferase (LUC) activity was normalized with *Renilla* luciferase (RLUC) activity. Results are representative of three independent experiments. Data shown are mean ± SD (n=3). All data were statistically evaluated using one-way ANOVA: **, 0.005< P < 0.01; ***, P < 0.005.
