# Supplementary material for: Identification of GA20ox2 as a target of ATHB2 and TCP13 during shade response
Source: Front Plant Sci. 2023 Apr 21;14:1158288. doi: 10.3389/fpls.2023.1158288 (PMC10160606; doi:10.3389/fpls.2023.1158288)
Supplement: Supplementary file 3 [file DataSheet_3.pdf]

Supplementary table

Son et al., Table S1.

Gene name	Primer name	DNA sequence
Real-time quantitative PCR		
GA20ox1	GA20ox1-RT-F	5’-CTTCCATCAACGTTCTCGAGC-3’
GA20ox1-	GA20ox1-RT-R	5’- GGTTTTGAAGGTCGATGAGAGG-3’
GA20ox2	GA20ox2-RT-F	5’-AGAAACCTTCCATTGACATTCCA-3’
GA20ox2	GA20ox2-RT-R	5’-AGAGATCGATGAACGGGACG-3’
GA3ox1	GA3ox1-RT-F	5’- ACTCGTCTCAAAGGCTGCAAC-3’
GA3ox1	GA3ox1-RT-R	5’- GAGGCTCTCATCGACACCATG-3’
ACTIN2-	ACTIN2-RT-F	5’-TCAGATGCCCAGAAGTCTTGTTCC-3’
ACTIN2	ACTIN2-RT-R	5’-CCGTACAGATCCTTCCTGATATCC-3’
EF1α	EF1α-RT-F	5’-TGAGCACGCTCTTCTTGCTTTCA-3’
EF1α	EF1α-RT-R	5’-GGTGGTGGCATCCATCTTGTTACA-3’
Yeast two-hybrid		
GAF1	GAF1-BamHI-F	5’-CTTCTCATACACGGAACCCTTCTG-3’
GAF1	GAF1-XhoI-R	5’-GCATTGCTCAGTACAAAGCTGGTG-3’
TCP13	TCP13-EcoRI-F	5’- AATGAATTCATGAATATCGTCTCTTGGAAA-3’
TCP13	TCP13-PstI-R	5’-CCCCTGCAGTCACATATGGTGATCACTTCC-3’
TCP13	TCP13-178aa-R	5’-GGGCTGCAGTCAAGTAGTAGACTTTTGTACAC-3’
TCP13	TCP13-179aa-F	5’-GGGGAATTCACTAGCCGCGAAGAAAACGC-3’
TCP13	TCP13-200aa-F	5’-GGGGAATTCCATGTTGGGTCTTATGGAAC-3’
TCP13	TCP13-280aa-R	5’-GGGCTGCAGTCAGCTTACCATTTGCCTCGGAT-3’
Dual luciferase assay		
GA20ox2	$P_{GA20ox2}$-2.0-F	5’-ACGCGTCGACGGAAATATAGACATTTTTATGCAC-3’
GA20ox2	$P_{GA20ox2}$-1.5-F	5’-ACGCGTCGACTTCATCCATTAAATAGTAATTC-3’
GA20ox2	$P_{GA20ox2}$-1.0-F	5’ACGCGTCGACGCTTTCTCTTATTTCATTTTCTTTGCG-3’
GA20ox2	$P_{GA20ox2}$-0.5-F	5’ACGCGTCGACTGGAAAACATAAAGAATTTTAAGATG-3’
GA20ox2	$P_{GA20ox2}$-0.2-F	5’-ACGCGTCGACAAGATTTTGTTCAATATACAC-3’
GA20ox2	$P_{GA20ox2}$-SalI-R	5’-CCCCCCGGGTTCTTTTCTTTTTTTTTTCTTGAGAG-3’
GA20ox2	$P_{GA20ox2}$(1.5-1.0kb)-F	5’-TTTGTCGACTTCATCCATTAAATAGTAATTC-3’
GA20ox2	$P_{GA20ox2}$(1.5-1.0kb)-R	5’-TTTCCCGGGATTAGTATTTCAGTAAATATTAAC-3’
GA20ox2	$P_{GA20OX2}$(ABS1m)-F	5'-CAATTTGAAGAACCGTGTAGGAATTAGTTAC-3'
GA20ox2	$P_{GA20OX2}$(ABS1m)-R	5'-ACTAATTCCTACACGGTTCTTCAAATTGGAA-3'
GA20ox2	$P_{GA20OX2}$(ABS2m)-F	5'-ATACATGACCGTCGTGCAAGATTCCGATAAG-3'
GA20ox2	$P_{GA20OX2}$(ABS2m)-R	5'-AGAATCTTGCACGACGGTCATGTATATATTG-3'
VP16	VP16-KpnI-F	5'-GGGGATGGTACCGCCCCCCCGACCGATGTCAGC-3'
VP16	VP16-BstBI-R	5'-AAAGTATTCGAATTACCCACCGTACTCGTCAAT-3'
Transgenic plant		

ATHB2	pTA7002-ATHB2-GFP-F	5'-AAACTCGAGATGATGTTCGAGAAAGACGAT-3'
GFP	pTA7002-ATHB2-GFP-R	5'-CCCACTAGTTTATTTGTATAGTTCATCCAT-3'
EMSA		
GA20ox2	$P_{GA20OX2}$ (ABS1)-F-Biotin	5'-CAATTTGAAGACAATGTTAGGAATTAGTTAC-Biotin-3'
GA20ox2	$P_{GA20OX2}$ (ABS1)-R-Biotin	5'-GTAACTAATTCCTAACATTGTCTTCAAATTG-Biotin-3'
GA20ox2	$P_{GA20OX2}$ (ABS1)-F	5'-CAATTTGAAGACAATGTTAGGAATTAGTTAC-3'
GA20ox2	$P_{GA20OX2}$ (ABS1)-R	5'-GTAACTAATTCCTAACATTGTCTTCAAATTG-3'
Chromatin immunoprecipitation (ChIP) assay		
GA20ox2	Ch-GA20OX2-F1	5'-TGGGGCCTTTGTAGCTATTG-3'
GA20ox2	Ch-GA20OX2-R1	5'-TGAATCCGAAACGAAAATCG-3'
GA20ox2	Ch-GA20OX2-F2	5'-TTAAAAACAAATATCACATATCATTCG-3'
GA20ox2	Ch-GA20OX2-R2	5'-TCATTACTCGCATTTATTTTTGTAA-3'
GA20ox2	Ch-GA20OX2-F3	5'-TGCAAAATCGACCCCATATC-3'
GA20ox2	Ch-GA20OX2-R3	5'-GTTCCCGAGGTTGTGTTTTG-3'
GA20ox2	Ch-GA20OX2-F4	5'-CCATGGCAATTTGTTTGTTG-3'
GA20ox2	Ch-GA20OX2-R4	5'-TTTTGGCCAACATCTTAAAATTC-3'
GA20ox2	Ch-GA20OX2-F5	5'-GGTCATACATTCTAAAACTTTTGTGG-3'
GA20ox2	Ch-GA20OX2-R5	5'-TGAACAAAATCTTTGGTCTCAATC-3'
GA20ox2	Ch-GA20OX2-F6	5'-TGATTGCAAGATTCCGATAAGA-3'
GA20ox2	Ch-GA20OX2-R6	5'-GAGGAGATGGGGGAACAAAG-3'
PP2A	PP2A_ChIP_F	5'-CTGGCGTGTGCGTTATATG-3'
PP2A	PP2A_ChIP_R	5'-CAAACATGGACTTCCAAGTACC-3'

Table S1. Primers used in this study
